# Modelling esophageal adenocarcinoma and Barrett’s esophagus with patient-derived organoids

**DOI:** 10.3389/fmolb.2024.1382070

**Published:** 2024-04-24

**Authors:** Julia V. Milne, Ebtihal H. Mustafa, Nicholas J. Clemons

**Affiliations:** ^1^ Division of Cancer Research, Peter MacCallum Cancer Centre, Melbourne, VIC, Australia; ^2^ Sir Peter MacCallum Department of Oncology, The University of Melbourne, Melbourne, VIC, Australia

**Keywords:** esophageal adenocarcinoma (EAC), patient-derived organoid (PDO), Barrett’s esophagus, models of cancer, organoids, esophageal cancer (EC), tumorigenesis

## Abstract

Currently, esophageal adenocarcinoma (EAC) research is hindered by a dearth of adequate models to study this disease. Traditional cell line and genetically engineered mouse models are lacking in biological and physiological significance, whilst the inefficiency of patient-derived xenografts limit their potential applications. This review describes the landscape of EAC research using patient-derived organoids (PDOs). Here, we detail the methods of establishment and optimization of EAC PDO cultures, as well as current and prospective applications of these models. We further highlight a crucial knowledge gap in the mechanisms of EAC transformation from its precursor lesion, Barrett’s esophagus (BE). As such, we also describe the culture requirements of BE PDOs and attempts to model tumorigenesis using PDO models.

## 1 Introduction

Esophageal cancer (EC) is a leading cause of cancer-related deaths worldwide, accounting for over half a million deaths and approximately the same number of new diagnoses annually (Bray et al., 2018; Ferlay et al., 2021). Despite some advances in detection techniques and therapies, prognosis following an EC diagnosis is dismal and the 5-year survival probability remains less than 20%. (Uhlenhopp et al., 2020). The dire nature of esophageal cancer may be attributed to the high frequency of late diagnoses and an insufficient understanding of the molecular drivers of this malignancy, leading to a lack of targeted therapies. Robust preclinical models to study EC are therefore essential to enhance our knowledge of this devastating disease and improve outcomes for patients.

Two major subtypes constitute the majority of EC cases: esophageal squamous cell carcinoma (ESCC) and esophageal adenocarcinoma (EAC). ESCC and EAC are distinct in their histology, classic anatomical location, risk factors and geographic distribution. ESCC is the most common subtype observed globally, constituting approximately 87% of all esophageal cancer cases, and is most strongly associated with history of tobacco smoking and alcohol consumption (Arnold et al., 2015; Arnold et al., 2017; Huang and Yu, 2018; Ilson and van Hillegersberg, 2018). Rates of ESCC are declining, likely due to the reduction in smoking (Bray et al., 2018), and survival rates are growing with improved treatment regimens implemented since 1973 (Njei et al., 2016). In contrast, rates of EAC, now the predominant subtype in the USA, Europe and Australia, have been increasing exponentially for the past few decades and are expected to continue on this trend (Arnold et al., 2017; Ferlay et al., 2021). EAC currently makes up 11% of global esophageal cancer diagnoses (Arnold et al., 2015). Men over the age of 65 are at greatest risk of EAC (Bray et al., 2018), whilst other powerful risk factors for EAC include obesity, smoking, history of gastro-esophageal reflux (GERD) and the presence of the precursor lesion, Barrett’s esophagus (BE) (Arnold et al., 2017; Bray et al., 2018).

It is widely accepted that EAC develops from BE (Killcoyne and Fitzgerald, 2021). A consequence of chronic GERD, BE is characterised by gastric or intestinal metaplasia of the esophagus, where the normal squamous epithelium of the esophagus is replaced by segments of columnar mucosa, often emanating from the gastroesophageal junction (GEJ) (Que et al., 2019). BE frequently follows a linear sequence of progression, beginning as a non-dysplastic metaplasia before developing into low- and finally high-grade dysplasia (Killcoyne and Fitzgerald, 2021). Risk of progression to EAC increases with grade of dysplasia; patients with non-dysplastic BE have a 0.3% risk of developing EAC each year, and this increases to 5%–10% and 20% for low- and high-grade dysplasia respectively (Hvid-Jensen et al., 2011). The transition from BE to EAC, however, remains poorly understood. Therefore, elucidating the mechanisms of EAC development can provide a better understanding of the disease and improve the efficiency of early detection and intervention measures.

To date, most studies into the biology of EAC has been conducted using cancer cell lines, genetically engineered mouse models (GEMMs), and patient-derived xenografts (PDXs) (Liu et al., 2016; Mahmoudian et al., 2021; Liang et al., 2023). Cell lines have the advantages of being relatively cost effective, readily accessible and allowing for large-scale investigations (Kamb, 2005; Cheon and Orsulic, 2011). However, these models are notoriously poor at recapitulating real physiological conditions, particularly regarding responses to therapy, due to the artificial nature of cell culture conditions and lack of heterogeneity, while GEMMs are limited by inherent differences between human and murine physiology (Kamb, 2005). The immortalization process required to generate cell lines often brings about molecular changes (Underwood et al., 2010) or results in cell lines derived from single clonal populations (Garcia et al., 2016). The multistep progression of BE to EAC is notoriously difficult to model using GEMMs because BE does not naturally occur in rodents (Kapoor et al., 2015). On the other hand, PDXs offer a more accurate representation of human disease, but their limitations lie in the inefficiency of engraftment as well as time and resources (Kamb, 2005). More recently, the development of techniques for culturing patient-derived organoids (PDOs) has served to bridge the gap between traditional *in vitro* cell models and PDXs. This review discusses the emerging landscape of EAC research using PDO models.

## 2 Establishment and optimization of EAC patient-derived organoid cultures

### 2.1 Derivation of EAC organoids from patient tissue

Over a decade ago, a seminal study demonstrated reliable and reproducible culturing of three-dimensional structures that mimicked the configuration and self-organizing phenotype of mammalian intestinal crypts (Sato et al., 2009a). These structures, named “organoids,” were derived from intestinal stem cells, and were shown to differentiate into the complex architecture of the crypts and villi of the small intestine. Since then, organoids have been derived from gastrointestinal (Sato et al., 2009a; Jung et al., 2011; Sato et al., 2011; Boj et al., 2015), reproductive (Kessler et al., 2015; Turco et al., 2017) and many other tissue origins with epithelial, glandular and neoplastic phenotypes (Karthaus et al., 2014; Maimets et al., 2016; Sachs et al., 2018). Organoids have been shown to replicate the structural complexity of many tissue types, as well as recapitulate differentiated cellular functions and expression profiles of the donor tissue (Sato et al., 2011; Yan et al., 2018; Kopper et al., 2019; Mazzucchelli et al., 2019). As such, organoids may be considered a more appropriate model system for studying human disease.

Recent studies have sought to establish organoids derived from EAC patients (Li et al., 2018; Karakasheva et al., 2020; Karakasheva et al., 2021), adopting methodologies inspired by those used in gastrointestinal organoid culture (Sato et al., 2011; van de Wetering et al., 2015; Broutier et al., 2016). Despite the relative newness of these protocols compared to PDXs, the success rates for EAC PDO establishment have proven to be greater than that of PDXs (Liu et al., 2018; Liang et al., 2023). The PDO derivation process involves mechanically fragmenting and enzymatically digesting EAC tissue specimens (diagnostic biopsies or surgically resected tumor tissues), followed by seeding cells into or onto a 3D extracellular matrix (e.g., Matrigel) and propagating them through successive passaging rounds (Li et al., 2018; Karakasheva et al., 2020; Karakasheva et al., 2021). Digestion media typically include a combination of collagenase, dispase and hyaluronidase to obtain a single cell suspension and remove non-tumor contaminants such as stroma (Karakasheva et al., 2020; Du et al., 2022). Given the anatomical location of the tumors and association with food matter, EAC primary cultures are particularly susceptible to contamination by bacteria and fungi. It is therefore essential to pre-treat or wash biopsy specimens with antimicrobial agents. To this end, Karakasheva *et al.* recommend extensive rinsing with saline, broad spectrum antibiotics (e.g., a cocktail of penicillin/streptomycin, gentamicin, and amphotericin B) and short-term storage in organ transplant preserving solution (34).

Li et al. stand as pioneers in detailing the creation of human EAC organoids from surgically resected human tissue, reporting an overall success rate of 31% (10 out of 32 samples) (Li et al., 2018). The authors highlighted that the primary reasons for culture failure in ∼70% of the samples were insufficient growth from the initiation of the culture and issues related to contamination, both of which have since been observed by other groups (Derouet et al., 2020). Intriguingly, recent studies demonstrated that addition of certain supplements (i.e., CHIR99021 [Wnt activator], Gastrin, Y-27632 [anoikis inhibitor], and N2 supplements) to the culture medium significantly increased the success rate to approximately 78% (7 out of 9 samples) (Karakasheva et al., 2020; Karakasheva et al., 2021). However, it is important to mention that in these later studies (Karakasheva et al., 2020; Karakasheva et al., 2021), EAC organoids were generated from endoscopic biopsies collected only from treatment-naive patients at the time of diagnosis, whilst Li et al. used samples collected from both treatment-naive patients and those who had undergone chemotherapy (Li et al., 2018). This could potentially explain the low success rate of organoid culture within this study, as chemotherapies are known to induce various alterations in tumor tissues, affecting the viability and growth capacity of cells. Unfortunately, the lack of specific details in the methodology of this paper prevents us from determining the percentage of successfully grown organoids within each patient group. Additionally, whilst one detailed EAC-specific protocol exists for organoid derivation from patient tissue (Karakasheva et al., 2020), research groups apply their own modifications to various protocols and, as such, success rates are variable between studies.

### 2.2 Culture media for EAC PDOs

The culture of EAC PDOs utilizes a basal medium composed of advanced Dulbecco’s Modified Eagle Medium (DMEM)/F12, supplemented with antimicrobial agents (penicillin/streptomycin or imipenem/cilastatin), Hanks’ Balanced Salt Solution (HBSS), GlutaMAX, and antioxidant agents (N-acetylcysteine, N2, & B27) (Li et al., 2018; Karakasheva et al., 2020; Karakasheva et al., 2021). The basal medium is further enriched with key components of gastrointestinal organoid culture (Sato et al., 2009a; Sato et al., 2011; Schepers and Clevers, 2012) including Wnt signalling pathway activators (Wnt3a and R-spondin), a BMP signalling pathway inhibitor (Noggin), a TGF-β inhibitor (A83-01), a p38 inhibitor (SB202190), anoikis inhibitors (ROCK inhibitors), as well as epidermal and fibroblast growth factors (EGF and FGF10) (summarized in Table 1). These supplements are included in the protocol for culturing EAC PDOs because of the similarity between EAC and intestinal epithelium. While these supplements are important for cultivating intestinal organoids from normal intestinal tissues, it is yet unclear whether they are essential for growing cancer organoids, where self-sufficiency in growth signals and plasticity are distinctive features.

**TABLE 1:** Summary of culture conditions for EAC PDOs in published studies.

Study Culture conditions	Li et al. (2018)	Karakasheva et al. (2020)	Cruz-Acuña et al. (2023)
Source of organoids	Resected EAC tissue samples	Diagnostic biopsies or surgically resected OAC tissues	Tissue biopsies
**Basal medium**	aDMEM/F12	aDMEM/F12	aDMEM/F12
**Supplements**			
HEPES	1x	10 mM	10 mM
Glutamax/L-Glutamine	1x	1x	1x
Antimicrobial	1x Penicillin/streptomycin & 1 mg/mL Primocin	5 µM Gentamicin & 1x antibiotic-antimycotic	10 µM Gentamicin & 1x antibiotic-antimycotic
NAC	1 mM	1 mM	1 mM
B27	1x	1x	1x
N2	-	1x	1x
RSPO1-conditioned medium	20% (in volume)	20% (in volume)	50% L-WRN cell-conditioned medium (expressing Wnt-3A, R-Spondin1, and Noggin)
Noggin	100 ng/mL	100 ng/mL	
Recombinant Wnt-3A or Wnt-3A conditioned medium	50% (in volume)	50% (in volume)	
EGF	50 ng/mL	250 ng/mL	250 ng/mL
FGF10	100 ng/mL	100 ng/mL	-
Nicotinamide	10 mM	20 mM	20 mM
SB202190	10 μM	1 μM	1 μM
Gastrin	-	100 nM	100 nM
Y-27632	-	10 μM	10 μM
A83-01	100 nM	500 nM	500 nM
CHIR99021	-	100 nM	500 nM
**Digestion method**	A mechanical digestion followed by an enzymatic digestion using collagenase II (1.5 mg/mL) at 37°C for 1–2 h	Enzymatic digestion using dispase and trypsin-EDTA followed by mechanical dissociation	Enzymatic digestion using dispase and trypsin-EDTA followed by mechanical dissociation
**Matrix**	Cultrex BME	Matrigel	Matrigel

Most of these supplements influence pathways that are crucial for the self-renewal of human gastrointestinal stem cells (Mishra et al., 2005; Schepers and Clevers, 2012; Bartfeld et al., 2015; Broutier et al., 2016), mirroring *in vivo* modulation by the surrounding environment. The canonical Wnt/β-catenin pathway is recognized as a vital regulatory signalling axis that impacts developmental processes and governs the maintenance, self-renewal, and differentiation of stem cells in adult mammalian tissues (Schepers and Clevers, 2012). Indeed, using Wnt proteins, such as Wnt3a, along with the Wnt signalling potentiator, R-spondin, enhances plating efficiency, stem cell maintenance, and long-term survival of normal human small intestine (Sato et al., 2009a), colon (Sato et al., 2011), and gastric (Barker et al., 2010) organoids. Noggin and A83-01 are two inhibitors that prevent downstream signalling via the TGF-β pathway by inhibiting bone morphogenetic protein (BMP) and the TGF-β type I receptor, ALK5, respectively. Given that TGF-β signalling has an inhibitory effect on proliferation while promoting apoptosis and differentiation in esophageal epithelial cells (Osaki and Gama, 2013), the organoid culture medium incorporates the TGF-β inhibitor A83-01. This addition aims to enhance culture efficiency by counteracting the suppressive impact of TGF-β signalling. Moreover, research indicates that inhibiting BMP is essential for deriving esophageal epithelial progenitors from human pluripotent stem cells (Zhang et al., 2018). This emphasizes the importance of utilizing BMP antagonists to ensure the prolonged maintenance of stem cells in esophageal progenitor cells. However, the scenario may differ when considering EAC organoids, as studies propose that BMP signalling could play a significant role in the development of Barrett’s esophagus and EAC (Wang et al., 2010; Westra et al., 2022; Correia et al., 2023). Consequently, blocking BMP might not be deemed necessary for EAC PDOs.

One organoid medium component that has attracted controversy is SB202190, a small molecule pyridinyl imidazole p38 MAP kinase inhibitor that directly binds the ATP binding pocket of p38 MAP kinases (Costa et al., 2023). It has been shown by Sato and colleagues (Sato et al., 2011) that adding SB202190 to normal human colon mucosa organoids increased proliferation, extended culture periods (from 3 to 6 months) and maintained normal budding structure in these cells. In contrast, organoid formation by normal human squamous esophageal cells was hindered by SB202190 (Kasagi et al., 2018). Oxidative stress, potentially inducing apoptosis through p38 MAP kinase, may be a contributing factor (Zarubin and Han, 2005). Notably, the esophageal organoids in this study were cultured in medium lacking antioxidant supplements like N2, B27, insulin, transferrin, and selenium, suggesting that antioxidants could potentially mitigate cellular stress and enhance cell survival. In line with its observed anti-proliferative effect, Fujii et al., 2016 demonstrated the potential of SB202190 to reduce the proliferation of colorectal tumor organoids. This finding highlights the importance of refining the tumor organoid culture protocol based on the intricacies of tumor biology and differences between normal and tumor tissue requirements. Moreover, we know that aberrant driver pathways delimit the niche-restricted growth of cancer cells and allow them to grow out of control, leading to cell overgrowth and the ability to spread. Hence, it remains to be seen whether all these supplements and growth factors that sustain stemness and promote growth of organoids from non-transformed cells are necessary for tumor organoid culture. Additionally, it’s unclear whether the inclusion of these supplements could potentially have a counterproductive effect by selecting for a subset of tumour clones, thereby altering the heterogeneity within organoid populations. Consequently, it is necessary to understand the molecular and functional niche dependence of tumor organoids and adjust culture protocols accordingly. While this point has been addressed in the organoid culture of several cancer types such as ovarian (Kopper et al., 2019; Maenhoudt et al., 2020), colorectal (Sato et al., 2009a; Sato et al., 2011), breast (Mazzucchelli et al., 2019; Dekkers et al., 2021), and prostate (Drost et al., 2016), testing this in the context of esophageal adenocarcinoma has been limited. This is largely due to the interpatient heterogeneity of the disease, the presence of very few common molecular drivers, and therefore the absence of a comprehensive understanding of the biological niche of this cancer subtype. Thus, the precise requirements of EAC PDO cultures remain to be elucidated, and more work is required to determine whether specific components should be included or excluded from protocols.

### 2.3 Matrix scaffolds for EAC PDOs

While our primary emphasis in this discussion has been on the composition of the culture media as a key factor influencing the rate of organoid establishment, it is imperative to recognize that other variables, including the quality of sampling, sample digestion, matrix scaffold, and technical handling, also play a vital role. Although most of these factors have not been examined concerning their effects on EAC PDO culture, a recent study attempted to explore how altering mechanical and biochemical matrix properties could impact growth and mutational profile of EAC PDOs (Cruz-Acuña et al., 2023). Initially, this study demonstrated that varying the concentration of Matrigel resulted in changes in the growth and transcriptional expression of EAC-associated genes (e.g., *TP53*, *NOTCH1* and *ZEB1*). However, discerning whether these effects are mediated by differences in the mechanical properties (e.g., stiffness) or biochemical properties of Matrigel proves challenging. Matrigel batches exhibit variability in these properties, making it difficult to recapitulate the independent effects of each. To bridge this knowledge gap, Cruz-Acuña et al., 2023 designed a hydrogel matrix to mimic the extracellular matrix (ECM) found in EAC tumors. This matrix is constructed using hyaluronic acid (HA) and is specifically crafted to include adhesive peptides and protease-degradable peptide crosslinkers. By adjusting the densities of these two components, it becomes possible to control both cell/matrix interactions and the stiffness of the hydrogel, respectively. Interestingly, when EAC PDOs were cultured in HA hydrogels with mechanical properties varying from a “soft” matrix (similar to Matrigel) to a “stiff” matrix (resembling tumour ECM), there was a notable enhancement in organoid formation, size, proliferation and culture time within the stiff matrix. However, with a further escalation in stiffness, the organoids displayed a considerable decrease in formation, growth, and cell proliferation compared to the original stiff matrix. Mechanistically, EAC organoids cultured in the stiff hydrogel showed a significant increase in the expression and nuclear localization of Yap and SOX9 as compared with the organoids within the softer hydrogels or Matrigel. The Yap/SOX9 axis is known to activate cancer stem cell features and has demonstrated involvement in promoting the proliferation and metastasis of various cancer types, including gastrointestinal carcinoma (Song et al., 2014; Zanconato et al., 2016). Importantly, the overall mutational profile of the EAC PDOs did not change over a 14-day period, irrespective of the matrix type (Matrigel *versus* HA) or matrix stiffness. Overall, this study underscores the importance of tailoring matrix properties for culturing EAC PDOs to optimize survival and expansion times.

### 2.4 Validation of EAC PDOs and recapitulation of tissue of origin

In terms of histological, mutational, and gene expression profiles, EAC PDOs have been shown to faithfully replicate the characteristics of their primary tumors (Li et al., 2018; Derouet et al., 2020; Karakasheva et al., 2020; Karakasheva et al., 2021). Histopathological confirmation typically involves immunohistochemistry (IHC) and immunofluorescence staining of epithelial-specific and non-specific EAC markers such as cytokeratin 7 (CK7), mutant p53, and caudal type homeobox 2 (CDX2). Genomic profiling has confirmed the status of the main EAC drivers reported in the literature (e.g., *TP53*, *CDKN2A*, *a*nd *PIK3CA*) (Secrier et al., 2016a; Cancer Genome Atlas Research et al., 2017; Frankell et al., 2019) in EAC organoids, and mutations and copy-number alterations are consistent with patient-matched tumours. However, multiple studies have reported gene expression signatures or gene and protein alterations that were specific to either tumors or organoids (Li et al., 2018; Derouet et al., 2020). For example, Derouet et al., 2020 detected 78%–94% short tandem repeat (STR) concordance between tumor samples and early passage PDOs, but whole exome sequencing (WES) concordance was only 64.1% for high confidence single nucleotide variants (SNVs), whilst lower confidence calls were even more variably detected. Similarly, Li et al., 2018 report correlation coefficients ranging between 0.41 and 0.84 with regards to gene expression of matched patient tumour and PDO pairs. Such discordance might be explained by sub-clonal selection during organoid culture or outgrowth of non-tumor cells, as well as differences in the biological microenvironment that are not reflected in organoid culture.

One key advantage of PDO cultures over traditional cell lines is the capacity of organoid libraries, or biobanks, to mimic the diversity and heterogeneity of these characteristics amongst EAC patients. Derouet and colleagues histologically and genomically characterised a biobank of EAC organoids derived from pre-treatment endoscopic biopsies to investigate such inter-patient heterogeneity (Derouet et al., 2020). They found highly variable doubling time between PDOs, possibly representative of unequal growth rates of the parent tumor. Furthermore, they identified different *TP53* point mutations in each sample and low frequency of mutations shared between samples, signifying the inter-patient genetic diversity typical of EAC clinical specimens (Secrier et al., 2016a; Frankell et al., 2019). Histologically, the same group observed cytological similarity between matched biopsy-organoid pairs, where pairs exhibited a similar nucleus:cytoplasm ratio and either structured columnar or poorly differentiated appearance, but found that PDOs in general were quite architecturally homogenous (Derouet et al., 2020). This study encompasses both the benefits and limitations of organoid models in demonstrating inter-patient heterogeneity, where genomic, transcriptomic and cytological features displayed variation between samples, but EAC PDOs still lack in replicating the precise structural architecture of the original tissue.

Organoid libraries have also demonstrated the variety of possible responses to treatment regimes. The biobanks of EAC PDOs characterised by Karakasheva et al., 2021 and Li et al., 2018 displayed diverse responses to both chemo- and novel therapies, reflective of the high inter-patient variability in clinical responses to treatment. Moreover, both studies reported little overlap between gene expression profiles of organoid lines, further indicating the vast differences between EAC patients and highlighting the need for personalized therapeutic strategies. Overall, it is evident that biobanking and the emergence of PDO libraries will allow for standardized studies and comparisons across the highly heterogeneous populations of EAC patients in the future. The subsequent section will delve into the current and potential applications of EAC PDOs in basic and translational research.

## 3 Current applications of PDO models in EAC

### 3.1 Investigating tumor cell biology

**FIGURE 1:**
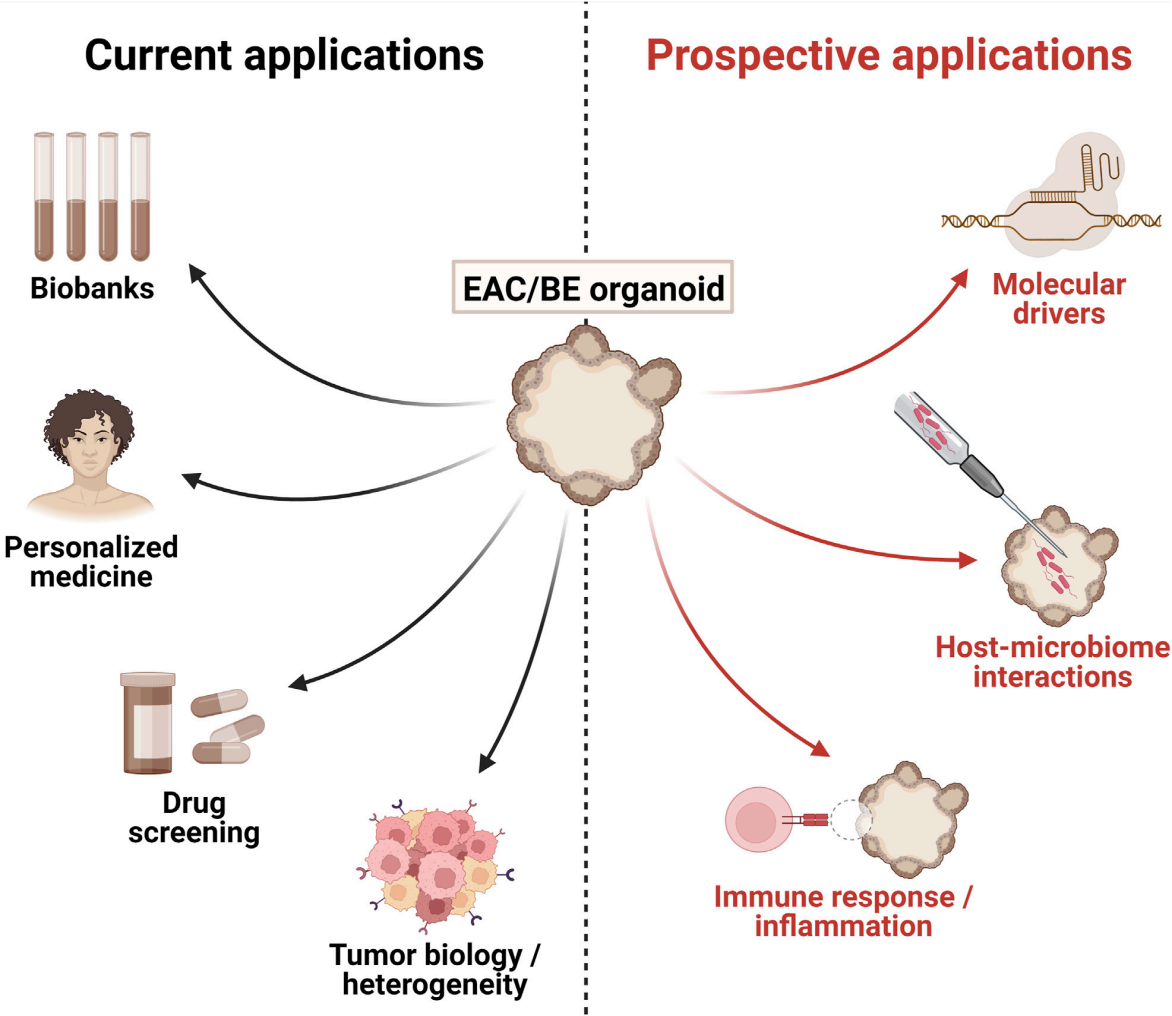
Current and prospective applications of patient-derived esophageal adenocarcinoma (EAC) and Barrett’s esophagus (BE) organoids. Figure generated using BioRender.

Biobanks of EAC PDOs have been utilised to study tumor cell biology and intra-tumoral diversity. In their seminal study, Li et al., 2018 *.* saw disrupted cell polarity in EAC PDOs, irregular cell structure and lack of organized apical/basal membranes resulting in multiple lumina. EAC PDO proliferation was also disordered, with Ki67 staining detected throughout the organoid mass, as opposed to emanating from the outer edges as in organoids from other cancer types (Sato et al., 2011), but consistent with staining patterns in EAC tumors (Li et al., 2018). This group also conducted spectral karyotyping to reveal that far from being clonal populations, organoids comprised clusters of aneuploid cells with variable chromosome numbers and structural rearrangements (Li et al., 2018). In line with the inter-patient diversity observed in EAC, the degree of karyotypic stability and amount of variation fluctuated between different PDOs. The authors were further able to track the clonal dynamics within organoid cultures, and reported clonal outgrowth of cells possessing pro-proliferative mutations such as in KRAS and diminution of clones lacking such mutations (Li et al., 2018). Therefore, investigation into the clonal evolution within EAC PDOs may reveal insights into niche factors and intra-tumoral clonal competition or co-operation (Figure 1).

EAC PDOs have also been applied in the study of cancer hallmarks. Cancer cell stemness has recently been considered a hallmark of cancer under the umbrella of “unlocking phenotypic plasticity” (Hanahan, 2022). The self-organizing, self-renewal and differentiation potential of organoids renders them an ideal model for investigating cancer stem cells. A recent study discovered a hypoxia-tolerant, CD44+/CD24-subpopulation within EAC PDOs that possessed stem cell properties and was enriched after exposure to γ-radiation (Du et al., 2022). Whilst it is difficult to compare stemness in organoids to that of cell lines, this finding points to a correlation between stemness and inherent resistance to radiation, consistent with that observed in EAC cell lines (Smit et al., 2013; Wang et al., 2014). The organoid-forming potential of EAC PDOs was increased when treated with Torin-1, an inhibitor of the mammalian target of rapamycin (mTOR), and reduced with mTOR stimulation with MHY1485, suggesting a role for mTOR in regulating stemness of cancer cells (Du et al., 2022). Together, this study presents a blueprint for using EAC PDOs to interrogate regulators of cancer cell stemness.

In sum, it is clear that EAC PDOs provide a rich platform and renewable resource for investigation into the genetic and phenotypic variations within a tumor, clonal evolution, extracellular dynamics and interaction between subpopulations.

### 3.2 Personalized medicine and patient avatars

Development of patient-matched therapeutic models such as PDOs allows for testing patient-specific drug combinations or personalized therapies by way of treatment avatars. Indeed, recent studies have demonstrated the capacity of EAC PDOs to predict patient response to first line therapies. Both Karakasheva et al., 2021 and Derouet et al., 2020 established organoid cultures from treatment naïve endoscopic biopsies and investigated response to standard-of-care neoadjuvant chemo- and radiotherapy regimens. Treatments tested included cisplatin, paclitaxel, 5-fluorouracil, epirubicin and γ-irradiation. In all cases, PDOs effectively mimicked the respective patients’ clinical response to each therapy. Interestingly, Derouet and colleagues reported that PDOs that displayed resistance to one drug were likely resistant to all standard-of-care therapies, indicating the potential value of pre-screening with PDOs in this way to prevent administering ineffective treatments to patients.

PDOs have also been utilised to screen second-line therapies in refractory or residual EAC. Li et al., 2018 derived organoids from post-treatment residual EAC tumors, which they tested against traditional chemotherapies. Perhaps unsurprisingly, they discovered poor sensitivity of the PDOs to standard-of-care therapeutics, in line with the matched patients’ poor clinical responses to neo-adjuvant therapy, and consistent with the first-line multidrug resistance observed by Derouet et al., 2020. As such, they used a biobank of nine PDOs to screen 24 additional compounds, including eight FDA-approved drugs and 10 pre-clinical targeted therapies (Li et al., 2018). Interestingly, this group observed consistency in drug sensitivity across consecutive PDO passages despite evidence of changing clonal dynamics (Li et al., 2018). They further conducted hierarchical clustering of samples based on sensitivity profile and found that PDOs clustered by genomic signature (e.g., DNA damage response [DDR] deficient, mutagenic, C>A/T dominant), where DDR deficient PDOs were sensitive to the most compounds. This study highlights the power of combining EAC PDOs and genomic profiling to inform future treatment decisions and the potential of non-traditional therapeutic pathways.

Whilst the relative speed of establishing cultures puts PDOs ahead of PDXs for the purposes of pre-treatment screening (Liu et al., 2016), the logistics of real-time prediction of drug response and subsequent implementation remain challenging. As detailed previously, success rates of EAC PDO derivation are imperfect, and the time from PDO generation to usability in medium-to-high throughput assays may be in the order of weeks (Bose et al., 2021). Additionally, sampling bias from biopsy specimens may result in incorrect representation of a heterogeneous tumor (Bose et al., 2021). Therefore, the use of PDOs as patient avatars for identifying effective treatments in a personalized fashion may not be a viable option. Arguably, a more reasonable and beneficial application of PDOs is the creation of well-characterized culture biobanks which can be used for trialing treatment regimens or drug screening (Song et al., 2014; Zanconato et al., 2016; Frankell et al., 2019). In this way, PDO biobanks may be employed to interrogate genotypic or phenotypic correlations with drug response that may inform future clinical trials.

### 3.3 Screening novel and targeted therapies

Targeted therapies for EAC are lacking, but application of PDOs for target discovery and validation may serve to improve productivity in this domain and hasten transition to the clinic. The HER2-targeted drug trastuzumab has been approved for clinical use in EAC, and recent publications have called attention to the effectiveness of anti-HER2 therapies in selectively targeting *ERBB2*-amplified EAC PDOs. After validating both genomic amplification and expression of the target protein in PDOs and matched biopsy specimens, Derouet et al., 2020 tested the efficacy of an alternative anti-HER2 compound, mubritinib, across their PDO biobank. Cell death was observed only in the one PDO that overexpressed HER2. Similarly, in a study by Vlachogiannis et al., 2018, the only *ERBB2*-amplified PDO was the most highly sensitive to the dual HER2/EGFR inhibitor lapatinib, again demonstrating drug selectivity and the promise of anti-HER2 therapies for treating *ERBB2*-amplified EAC.

Other researchers have tested the suitability of novel and alternative therapies for EAC using PDOs. For example, pre-clinical small molecule inhibitors targeting STAT3 and MEK1/2, as well as proton beam therapy (currently in clinical trials for use in EAC), have shown variable efficacy in EAC PDOs (Karakasheva et al., 2021). Vlachogiannis et al., 2018 provided evidence that large-scale libraries can be used for high-throughput screening and personalized medicine applications. This group screened a library of 55 drugs currently in clinical use or in clinical trials, including targeted therapies, on a biobank of metastatic gastroesophageal cancer PDOs. Genomic analyses were conducted in parallel to identify biomarkers of response or resistance. This work is unique in its application of metastasis-derived PDOs, where models in other studies represent the primary disease. Given the lack of effective treatment options and the dire prognosis of metastatic EAC, the implications of these findings are especially pertinent. Together, these investigations lay the foundations for future work in identifying and validating therapeutic targets for precision medicine using EAC PDOs to target both early stage and metastatic disease.

### 3.4 Drug resistance mechanisms

The scalable nature of PDO culture renders these ideal models for exploring mechanisms of drug resistance. In their 2021 paper, Karakasheva et al., 2021 employed EAC PDOs as treatment avatars for chemotherapy, coupled with transcriptomic analyses. Transcriptional responses to therapy were found to cluster by PDO and not by treatment type, suggesting different resistance mechanisms at play that are dependent on patient characteristics. Indeed, they observed very little overlap between differentially expressed genes in PDOs treated with cisplatin or paclitaxel, with only some similarity in enrichment of cell cycle and DNA damage response pathways (Karakasheva et al., 2021). However, this study did not delve deeper into the exploration of potential mechanisms of resistance nor any novel therapies. Whilst an interesting concept, this work only included three PDO lines. Thus, a larger biobank is likely required to determine transcriptional responses to treatment that may be globally relevant. Another study examined therapy response in heavily pre-treated metastatic gastroesophageal cancer PDOs (Vlachogiannis et al., 2018). This work reported that PDOs derived from refractory tumors were significantly less sensitive to first-line chemotherapies than their baseline counterparts, demonstrating acquired resistance. In fact, the degree of resistance was in line with that exhibited by PDOs with primary paclitaxel resistance. Alas, this study failed to further investigate the possible mechanisms of resistance, and this remains an intriguing future direction for PDO research.

The above represent the few examples of current applications of EAC PDOs. Unfortunately, existing studies all possess relatively low sample sizes, ranging from five to eight individual patients per study (Li et al., 2018; Derouet et al., 2020; Karakasheva et al., 2021). Therefore, the authors are unable to determine statistical significance or extrapolate their findings, but instead point to promising trends. However, as the technology continues to advance and become more accessible, PDOs are poised to revolutionize our understanding and treatment of this aggressive cancer. In the meantime, the potential for EAC PDOs may be realized by following the trails blazed in other cancer types.

## 4 Prospective uses for EAC PDOs

PDOs represent a unique model for interrogating the interactions between tumor cells and a modifiable microenvironment. Co-culture of PDOs with stromal or immune cells allows researchers to investigate the role of the microenvironment in tumor progression and resistance to therapy. Recent advances in immunotherapies have seen the application of immune checkpoint inhibitors (ICIs) as standard-of-care treatment for EAC (Kelly et al., 2021; Obermannová et al., 2022). Nivolumab, an anti-programmed cell death protein 1 (PD-1) antibody ICI, is indicated both in the adjuvant setting following chemoradiotherapy and for metastatic EAC (Obermannová et al., 2022). Interestingly, this indication is not dependent on expression of programmed death-ligand 1 (PD-L1) as is the case with use of nivolumab in advanced ESCC. This contradiction suggests it is necessary to improve our understanding of the interaction of EAC with the immune microenvironment to better stratify patients for ICI therapy. Neal et al., 2018 developed a PDO-based air-liquid interface methodology that preserved the architecture of the source tumor as well as much of the stroma from multiple cancer types. Using this model, the authors reproduced *in vivo* tumor-infiltrating lymphocyte (TIL) responses to nivolumab, including activation, expansion and tumor cell killing phenotypes. Other groups have demonstrated the viability of co-culturing immune cells (e.g., CD8^+^ T cells) with tumor organoids and identified correlation between patient and PDO responses to ICIs and other immunotherapies (Dijkstra et al., 2018; Forsythe et al., 2021; Yu et al., 2021; Kholosy et al., 2022). Such approaches may be utilized with EAC PDOs to better comprehend the interplay between the immune microenvironment and EAC tumors.

In a similar vein, EAC PDOs provide a novel platform for investigating host-microbiome interactions. The GI tract houses an abundance of microorganisms, known collectively as the microbiome, that influence normal biological functions as well as the pathology of cancer (Matson et al., 2021; Sepich-Poore et al., 2021). The microbiome has been shown to affect epithelial cell proliferation, inflammation and immune responses, as well as inducing mutagenesis in human cells (Matson et al., 2021; Sepich-Poore et al., 2021). For example, *H. pylori*, a common pathological bacterium that colonizes the stomach, is a well-characterized contributor to the progression of gastric dysplasia and neoplasia (Amieva and Peek, 2016; Moss, 2017). In contrast, EAC occurrence is negatively correlated with *H. pylori* infection (Polyzos et al., 2018), presenting an interesting paradox that warrants further investigation. However, attempts to study the interactions between *H. pylori* and human tissues *in vitro* have been fraught with difficulty. When co-cultured with gastric cell lines in monolayer, *H*. *pylori* undergo cell death upon attachment to the human cells (Segal et al., 1996; Kusters et al., 1997). Promisingly, patient-derived gastric organoid models allow for longer-term *in vitro* culture of *H. pylori* with PDOs, facilitating and accelerating research in this field (Chakrabarti and Zavros, 2020; Idowu et al., 2022). It stands that no equivalent studies exist using EAC PDO models to interrogate host-microbiome interactions, highlighting a clear gap in EAC research and a novel application of EAC PDOs.

When co-culturing stromal cells or microorganisms with PDOs, logistical complexity arises from the fact that the apical surface of cells is within the lumen of the organoid structure (Porter et al., 2020). Therefore, representative co-culture of bacterial or other cell populations with organoids requires microinjection of cultures to the interior of organoids, as opposed to their addition to the culture medium (Bartfeld et al., 2015; Hill et al., 2017; Puschhof et al., 2021). The lumen of gastric and intestinal organoids presents a hypoxic microenvironment, characterized by a mucus layer and an enclosed lumen, that supports the growth of commensal and pathogenic anaerobic bacteria (Bartfeld et al., 2015; Bartfeld, 2016). As esophageal epithelia, both normal and cancer-associated, also produce mucins (Stanforth et al., 2021), it may be assumed that EAC organoids are similar to other gastrointestinal organoids in this way. However, the presence of mucins in existing EAC PDO biobanks has not been confirmed. Unsurprisingly, microinjection also requires precise technical skills and limits the throughput capacity for co-culture experiments. Alternatives to microinjection involve manipulation of cells to reverse the apical-basolateral polarity of cells (Co et al., 2021; Kakni et al., 2023) or re-seeding of organoids as monolayers to expose the apical surface (VanDussen et al., 2015). The former of these is technically challenging whilst the latter results in loss of 3D structure and organization, reducing the physiological relevance of the model. Despite the limitations, the use of EAC PDOs for the study of host-microbiome interactions and the immune microenvironment is an exciting future prospect.

## 5 Modelling EAC tumorigenesis—the future of PDOs?

### 5.1 Models of EAC tumorigenesis

Studies of EAC tumorigenesis are limited by the lack of adequate models available to study the development and progression of BE to invasive disease. Whilst rodents are not known to naturally develop BE, exposure to acid bile has been shown to artificially induce a “Barrett’s-like” cell population in the mouse esophagus, but this is physiologically distinct from human BE (Quante et al., 2012; Lee et al., 2017). Canine and porcine models may be more anatomically relevant (Van Nieuwenhove and Willems, 1998; Krüger et al., 2017), but use of these models is costly, time consuming, and relies on heavy use of larger mammals and the ethical considerations that accompany these. Our group established a reliable model of progression of human dysplastic BE to EAC, by way of CRISPR/Cas9-edited cell line xenografts (Gotovac et al., 2021). However, this model has its own limitations. The microenvironment of the sub-cutaneous space, where BE xenografts are injected, is vastly different from that of the distal esophagus (Lee et al., 2018). Also, in comparison to tissue, cell line cultures are less heterogeneous and poorly recapitulate the original tissue (Kamb, 2005; Lee et al., 2018). Evidently, these insufficient models lead to a bottleneck in the study of BE progression to EAC.

### 5.2 PDO tumorigenesis models in gastrointestinal cancers

Organoid models of tumorigenesis in in gastrointestinal cancers have provided insight into the changing architecture of transforming structures and have proven to be vastly useful in studying mechanisms of neoplastic progression (Matano et al., 2015; Tao et al., 2019; Huang et al., 2020). PDOs offer a more cost-effective model than GEMMs to interrogate driver-specific tumorigenesis (Cheon and Orsulic, 2011; Mahmoudian et al., 2021). However, despite their seemingly ideal suitability to the task, organoid models have rarely been used to study the transition of BE to EAC. In other cancer types, biobanks of matched normal or pre-malignant tissue organoids with cancer organoids allow for functional examination of oncogenic progression (Jin and Mills, 2020). When compared to PDOs derived from normal tissue, cancer PDOs exhibit molecular changes that signal a transformation event has occurred. These changes may include the ability to thrive without supplementation of growth factors, loss of responsiveness to growth suppressive factors, limitless replicative potential and cell death evasion, anchorage-independent growth, expression of tumor-specific markers, or tumorigenic potential *in vivo* (Jin and Mills, 2020).

In this vein, PDOs have been used to study driver mutations and their functional consequences with regards to oncogenesis in GI cancers. Similarly to EAC, colorectal cancer (CRC) follows a sequence of progressive transformation, where normal intestinal tissue acquires mutations to develop into adenoma before evolving into invasive carcinoma (Vogelstein et al., 1988). Two key studies induced genetic knockout or point mutation of important CRC driver genes in human intestinal organoids (Drost et al., 2015; Matano et al., 2015). Matano et al., 2015 utilized electroporation whilst Drost et al., 2015 used a lipid-based system to transfect organoids derived from normal human intestinal epithelium with CRISPR/Cas9 gene editing machinery. Both groups generated *APC*, *TP53*, and *SMAD4* knockouts and constitutively active *KRAS* mutant (G12D or G12V) organoid lines, and Matano *et al.* also included a *PIK3CA*-activating point mutation (E535K). Modification of these driver genes lead to constitutive activation or inhibition of their relevant oncogenic or tumor suppressive pathways, respectively, eliminating the need for external stimulation or suppression. Therefore, edited PDOs were selected for via manipulation of these niche factors in the growth medium: removal of Wnt and R-spondin (to select for *APC* knockouts), nutlin-3 treatment (*TP53*), withdrawal of noggin and A83-01 and addition of TGF-β or BMP4 (*SMAD4*), removal of EGF or treatment with an EGFR inhibitor (*KRAS*), and a MEK inhibitor (*PIK3CA*). As a result, all multiple-modified PDOs were capable of proliferating in growth medium lacking all the aforementioned factors. Interestingly, despite engineering PDOs to possess the same genotypes, these two studies report significantly discordant findings. Drost et al., 2015 describe onset of tumor-like properties in quadruple modified (*APC* ^−/−^, *TP53* ^−/−^, *SMAD*4^−/−^, *KRAS* ^G12D^) normal organoids, which displayed an increase in mitotic errors and resultant aneuploidy, and grew into large, highly proliferative, and irregular tumors when xenografted into mice. In contrast, even the quintuple modified (*APC* ^−/−^, *TP53* ^−/−^, *SMAD*4^−/−^, *KRAS* ^G12V^, *PIK3CA* ^E535K^) organoids engineered by Matano et al., 2015 maintained normal ploidy and gene copy number, and a transcriptional profile that more resembled that of adenoma samples than CRC. Upon xenograft into the kidney capsule of mice, resultant nodules mirrored adenoma in both histological structure and metastatic potential. These data suggest that the PDOs engineered by Matano et al., 2015 had evolved adenoma-like characteristics, whilst Drost et al., 2015 cite the same genetic variations induced *bona fide* tumorigenesis of previously normal organoids. It must be noted that the sample sizes for both of these works are very small; only one PDO line was investigated by Drost and colleagues (Drost et al., 2015), whereas Matano *et al.* replicated their findings in a second PDO line and confirmed the observed phenotypes using multiple orthogonal approaches (Matano et al., 2015). The differences observed by these groups highlight the complexity of oncogenesis and the importance of large samples sizes and multiple measures to validate findings.

As CRC is known to arise after development of precursor lesions, Matano et al., 2015 extended the above findings by genetically modifying adenoma-derived PDOs. This group knocked out *TP53* and *SMAD4* and induced an activating mutation in *KRAS* or *PIK3CA* to model the final transition step of adenoma to carcinoma. When on a background of chromosomal instability, these triple modified adenoma PDOs displayed a highly irregular dysplastic phenotype and similar metastatic potential to CRC. These findings show that extensive changes occur in the normal-adenoma transition that are not adequately accounted for in both Drost et al. and Matano et al.’s work modelling tumorigenesis with normal PDOs as a starting material. Thus, it is more physiologically relevant to model oncogenic transformation using PDOs derived from pre-malignant tissue, such as adenoma or, in the case of EAC, BE. Like CRC, progression of BE to EAC is not monogenetic, so multiple gene manipulations would be required to model transformation. However, given the reported low success rates of gene editing in PDOs, this means multiple steps of clonal expansion would be required and would result in further deviation from the origin tissue. This is evident in both spotlighted studies, where extremely low efficiency of CRISPR\Cas9 editing was reported, and this may further explain the discordant results described by the authors.

### 5.3 Culturing Barrett’s esophagus organoids

A major factor contributing to the lack of PDO models of EAC tumorigenesis is the difficulty in establishing and propagating robust BE organoid cultures. First records of BE organoids appeared in 2011, when human metaplastic BE specimens were digested and seeded in Matrigel with a basal culture medium overlaid with gastrin, nicotinamide, A83-01, SB202190, Wnt-3A, noggin, EGF and R-spondin-1, leading to a successful passage over 1 month (5 passages) (Sato et al., 2011). However, for sustained long-term expansion beyond 20 passages, the addition of FGF10 to the culture medium became necessary (Sato et al., 2011). A subsequent study, using this same media, reported unsuccessful cultures for metaplastic BE specimens (Li et al., 2018). In contrast, Liu et al., 2018 achieved a success rate of 70% (7 out of 10 samples, with continuous propagation for up to 4–6 months) by following Sato et al., 2011 protocol with significant modifications, including the incorporation of prostaglandin E (PGE) and Y-27632. These modifications, known for preventing anoikis (Ishizaki et al., 2000; Joseph et al., 2005), were deemed essential for gastrointestinal (GI) organoid propagation (Sato et al., 2009a; Broutier et al., 2016). In recent and intriguing research, non-neoplastic, telomerase-immortalized human BE epithelial cell lines (BAR-T, BAR-10T) were used to explore organoid-like growth (Zhang et al., 2022). The study compared three distinct culture media: advanced DMEM/F12 (prepared according to the specifications outlined in Table 2), MCDB-153 (supplemented with hydrocortisone, insulin/transferrin, adenine, fetal bovine serum (FBS), bovine pituitary extract (BPE), L-glutamine, A83-01, & EGF), and DMEM/F12 (prepared like MCDB-153 but without BPE). Findings of this study indicated that organoid formation rates and viability were notably higher in advanced DMEM/F12 compared to the other two media (Zhang et al., 2022). This difference may be attributed to the presence of supplements in advanced DMEM/F12 that support stem cells and promote growth, which were lacking in the other two media. However, when applying advanced DMEM/F12 culture conditions to biopsy-derived Barrett’s epithelial cells, the success rate was around 10% (Zhang et al., 2022). Published culture conditions for BE PDOs are summarized in Table 2.

**TABLE 2:** Summary of culture conditions for BE PDOs in published studies.

Study Culture conditions	Sato et al. (2011)	Li et al. (2018)	Liu et al. (2018)	Zhang et al. (2022)	Cruz-Acuna et al. (2023)
**Basal medium**	aDMEM/F12	aDMEM/F12	aDMEM/F12	aDMEM/F12	aDMEM/F12
**Supplements**					
HEPES	10 mM	1x	10 mM	10 mM	10 mM
Glutamax/L-Glutamine	1x	1x	1x	1x	1x
Antimicrobial	1x Penicillin/streptomycin	1x Penicillin/streptomycin & 1 mg/mL Primocin	1x Penicillin/streptomycin & 1x Primocin	1x Penicillin/streptomycin	10 µM Gentamicin & 1x antibiotic-antimycotic
NAC	1 mM	1 mM	1 mM	1 mM	1 mM
Recombinant Wnt-3A or Wnt-3A conditioned medium	+	50% (in volume)	50% (in volume)	50% (in volume)	50% (in volume)
B27	1x	1x	1x	1x	1x
N2	1x	-	1x	1x	1x
RSPO1-conditioned medium	+	20% (in volume)	20% (in volume)	50% (in volume)	50% (in volume)
Noggin	+	100 ng/mL	100 ng/mL	50% (in volume)	50% (in volume)
EGF	+	50 ng/mL	50 ng/mL	50 ng/mL	250 ng/mL
FGF10	+	100 ng/mL	100 ng/mL	100 ng/mL	-
Nicotinamide	+	10 mM	10 mM	10 mM	20 mM
SB202190	+	10 μM	10 μM	10 mM	1 μM
Gastrin	+	-	10 nM	10 nM	100 nM
Y-27632	^a^10 μM	-	10 μM	10 mM	10 μM
A83-01	+	500 nM	500 nM	500 nM	500 nM
Prostaglandin E2	-	-	10 nM	-	-
CHIR99021	-	-	-	-	500 nM
**Digestion method**	^b^A mechanical digestion followed by incubation for 1 h at 37°C in a digestion buffer	A mechanical digestion followed by an enzymatic digestion using collagenase II (1.5 mg/mL) at 37°C for 1–2 h	The method was based on Sato et al. (2011)	A mechanical digestion followed by an enzymatic digestion using collagenase A (2.5 mg/mL) at 37°C for 1 h	Enzymatic digestion with dispase and trypsin-EDTA followed by mechanical dissociation and passing through a 100 μm cell strainer
**Matrix**	Matrigel	Cultrex BME	Matrigel	Matrigel	Matrigel

^a^ : Y-27632 was included in the medium for the first 2 days only.

^b^ : Digestive buffer components were not listed in the study.

+Concentration is not specified in the study.

As previously highlighted in this review, success in cultivating organoids relies not only on media composition but also on various factors such as matrix variation and digestion protocols. Unfortunately, these additional elements crucial to the organoid culture’s success often receive insufficient attention in the methodologies presented in the articles, posing a challenge in pinpointing the exact sources of variation. Nevertheless, histological analysis revealed that organoids successfully derived from endoscopic biopsies generally replicate characteristics of Barrett’s metaplasia (Sato et al., 2011; Liu et al., 2018; Zhang et al., 2022). Immunohistochemistry and fluorescent staining demonstrated that Barrett’s organoids exhibit Alcian blue staining typical of goblet cells, whilst also expressing Trefoil factor 3 (TFF3) as a goblet cell marker and mucin 2 (MUC2) as an intestinal columnar cell marker (Karthaus et al., 2014; Arnold et al., 2015; Kopper et al., 2019).

Together, these studies comprise the endeavors undertaken to cultivate Barrett’s organoids. However, they also emphasized a noteworthy inconsistency in the success of the cultures, revealing substantial variations in the results. This underscores the necessity for a thorough review and optimization of Barrett’s organoid establishment protocols to pinpoint the sources of variation and enhance the overall procedure.

### 5.4 PDO models of EAC tumorigenesis

Early works using PDOs to model neoplastic progression of EAC are lacking in efficiency, reliability, or biological significance. Liu et al., 2018 cultured BE PDOs as detailed in the above section and employed CRISPR/Cas9-mediated gene editing to knockout the *APC* gene. APC loss results in hyperactivation of the Wnt/β-catenin pathway; a common occurrence in EAC which is driven by *APC* mutation in approximately 10% of cases (Choi et al., 2000). To select for knockout populations, the authors withdrew Wnt-3A and R-spondin-1 from the growth medium, allowing for outgrowth of APC-deficient (and therefore constitutive Wnt-activated) organoids. From there, single surviving organoids were picked for clonal expansion. However, survival rates were extremely poor, with less than one viable PDO per well available for picking. This indicates an exceptionally low success rate for gene editing and highlights a major limitation to this study. Despite this, this work described numerous assays to measure phenotypic changes associated with neoplasia in PDOs. In line with the documented hallmarks of cancer (Hanahan, 2022), *APC*-deleted BE PDOs displayed increased proliferation and enhanced replicative capacity as the edited PDOs were able to be cultured for twice as long as control organoids (from five to 10 months) (Liu et al., 2018). *APC* knockout organoids also grew larger than their wildtype counterparts and exhibited more complex architecture, including a multilayered epithelium, atypical organization, multiple lumina and loss of polarity, similar to that observed in Li et al., 2018 EAC PDOs. Suppression of apoptosis, augmented nucleus size and increased mitotic activity also pointed to oncogenic transformation. An interesting phenotype observed in this study was a purported increase in collective migration in *APC* knockout BE PDOs (Liu et al., 2018). Measured sharp-edged protrusive strands emanating from PDOs embedded in a collagen I solution, as opposed to the more rigid Matrigel (Liu et al., 2018). Using time-lapse live-cell imaging, this study quantified the degree of protrusive migration as a proxy for cancer cell invasiveness. A subset of wildtype BE PDOs (−40%) initially demonstrated collective migration upon seeding into the collagen I microenvironment but retracted the protrusive strands over time. Conversely, −65% of APC knockout PDOs began to show extra-organoid extensions and 22% sustained them for multiple days, suggesting enhanced collective migration in this population and disrupted basement membrane integrity redolent of EAC.

In another recent study, Zhao et al., 2022 investigated the effect of various gene knockouts on normal gastroesophageal junction (GEJ) organoids. GEJ cancers and those of the gastric cardia are thought to be related to EAC (Nowicki-Osuch et al., 2021). This research found that deletion of *TP53* and *CDKN2A*, two genes frequently lost in EAC and GEJ adenocarcinoma, induced morphological features of dysplasia and neoplasia in previously normal PDOs (Zhao et al., 2022). Similar to Liu *et al.*’s findings with *APC* knockout BE PDOs (Liu et al., 2018), *TP53/CDKN2A* knockout GEJ PDOs exhibited more complicated organoid architecture, increased mitosis and enlarged nuclei, indicating induction of dysplasia (Zhao et al., 2022). Expression of TFF3 also accompanied the gene editing, suggesting initiation of intestinal metaplasia. Increased proliferation and organoid forming ability were also observed, and, conducive with neoplastic phenotypes reported by Liu et al., 2018, the dual knockout allowed for extended propagation and passage of organoids from six (for wildtype) to over 19 months (Zhao et al., 2022). Further, subcutaneous injection of *TP53/CDKN2A* dual knockout PDOs into immunocompromised mice resulted in tumor formation within 8 weeks, whilst unedited PDOs failed to form tumors after 5 months. The resultant tumors were reminiscent of gastroesophageal adenocarcinoma in their morphology and degree of differentiation, suggesting these gene knockouts were responsible for neoplastic transformation of the previously normal GEJ PDOs.

The aforementioned studies represent the only two known attempts at modelling EAC tumorigenesis with PDOs. These works highlight the difficulty in conducting gene editing on PDOs, particularly those derived from BE. Extremely low editing rates and the subsequent requirement to expand clonal populations from single organoids are undeniable weaknesses of such protocols. Additionally, both studies appear to make use of only PDO line, of either BE or GEJ origin, which was not otherwise characterised. Such small sample sizes reduce the potential significance of the findings. Also, given the spectrum of metaplasia-dysplasia in BE, it would be beneficial to confirm the p53 status of the PDOs to verify the degree of irregularity underlying the tissue sample. It is now understood that all cases of EAC arise from BE tissue (Theisen et al., 2002; Nowicki-Osuch et al., 2021). Thus, in modelling EAC tumorigenesis, it is necessary to use BE as a starting material as opposed to normal epithelial cells.

The possibility remains to use esophageal PDOs to model BE and driver-specific tumorigenesis, akin to traditional genetically engineered mouse models for studying tissue-specific cancer drivers. However, difficulties lie in determining phenotypes that definitively show that transformation has occurred in PDO models. The most common method for investigating tumorigenesis is xenotransplantation of organoid cultures into immunodeficient mice and monitoring tumor formation (Matano et al., 2015; Naruse et al., 2020; Zhao et al., 2022). However, in the case of EAC, such a read-out may not be ideal due to the anatomical differences between humans and mice that make orthotopic xenograft problematic, and the limited physiological relevance of sub-cutaneous injection. That EAC develops from BE, an already abnormal tissue type, adds an extra layer of complication. Dysplastic BE exhibits many cancer-like features, such as chromosomal abnormalities, increased proliferation and mutation of p53 (Secrier et al., 2016a; Newell et al., 2019), which make it difficult to distinguish from EAC *in vitro*. In fact, many protein markers have been identified that may distinguish between normal and BE tissue, but no consensus exists for reliable markers of EAC neoplasia (DiMaio et al., 2012; Kinra et al., 2018). Recent work has shown that tumor organoids develop a self-sufficiency to grow in culture without addition of growth factors, as well as loss of responsiveness to growth inhibition (Tao et al., 2019; Jin and Mills, 2020). As such, this is a possible avenue of exploration for determining tumorigenicity of genetically modified BE organoids.

## 6 Summary

This review summarizes the current landscape of PDO usage in EAC and proposes a multitude of possible future directions for this field. EAC PDOs have, to date, been used as models for personalized medicine and predicting treatment response, drug and radiation regimens, and potential resistance mechanisms. Large biobanks of EAC PDOs will be immensely useful in screening and validation of novel therapeutic agents, comprising a more clinically relevant platform for drug screening compared to traditional cell lines or animal models and facilitating the identification of promising therapeutic candidates.

In sum, current work on EAC PDOs provide important first steps in the development of a reliable and efficient models of PDOs. However, the field of EAC research is severely lacking a robust, physiologically relevant model for studying BE tumorigenesis. A major benefit of such a model would be the ability to study progression of BE to EAC, interrogating driver-specific phenotypes and interactions in real time, without the need for excessive use of animals. Whilst it is clear that further technological advancements are required, results in other cancer types are promising and PDOs may serve to fill the gap in this area of EAC research in future.

## References

[B1] Amieva M. Peek R. M. (2016). Pathobiology of Helicobacter pylori-induced gastric cancer. Gastric Cancer Gastroenterol. 150, 64–78. 10.1053/j.gastro.2015.09.004 PMC4691563 26385073

[B2] Arnold M. Laversanne M. Brown L. M. Devesa S. S. Bray F. (2017). Predicting the future burden of esophageal cancer by histological subtype: international trends in incidence up to 2030. Am. J. Gastroenterol. 112, 1247–1255. 10.1038/ajg.2017.155 28585555

[B3] Arnold M. Soerjomataram I. Ferlay J. Forman D. (2015). Global incidence of oesophageal cancer by histological subtype in 2012. Gut 64, 381–387. 10.1136/gutjnl-2014-308124 25320104

[B4] Barker N. Huch M. Kujala P. van de Wetering M. Snippert H. J. van Es J. H. (2010). Lgr5(+ve) stem cells drive self-renewal in the stomach and build long-lived gastric units *in vitro* . Stem Cell. 6, 25–36. 10.1016/j.stem.2009.11.013 20085740

[B5] Bartfeld S. (2016). Modeling infectious diseases and host-microbe interactions in gastrointestinal organoids. Dev. Biol. 420, 262–270. 10.1016/j.ydbio.2016.09.014 27640087

[B6] Bartfeld S. Bayram T. van de Wetering M. Huch M. Begthel H. Kujala P. (2015). *In vitro* expansion of human gastric epithelial stem cells and their responses to bacterial infection. Infect. Gastroenterol. 148, 126–136. 10.1053/j.gastro.2014.09.042 PMC4274199 25307862

[B7] Boj S. F. Hwang C.-I. Baker L. A. Chio I.In C. Engle D. D. Corbo V. (2015) Organoid models of human and mouse ductal pancreatic cancer Cell, 160, 324–338. 10.1016/j.cell.2014.12.021 25557080 PMC4334572

[B8] Bose S. Clevers H. Shen X. (2021). Promises and challenges of organoid-guided precision medicine. Med. Med. 2, 1011–1026. 10.1016/j.medj.2021.08.005 34617071 PMC8492003

[B9] Bray F. Ferlay J. Soerjomataram I. Siegel R. L. Torre L. A. Jemal A. (2018). Global cancer statistics 2018: GLOBOCAN estimates of incidence and mortality worldwide for 36 cancers in 185 countries. CA A Cancer J. Clin. 68, 394–424. 10.3322/caac.21492 30207593

[B10] Broutier L. Andersson-Rolf A. Hindley C. J. Boj S. F. Clevers H. Koo B. K. (2016). Culture and establishment of self-renewing human and mouse adult liver and pancreas 3D organoids and their genetic manipulation. Nat. Protoc. 11, 1724–1743. 10.1038/nprot.2016.097 27560176

[B11] Cancer Genome Atlas Research N. Working Group A. Asan U. Agency B. C. C. Brigham, Women's H. Broad I. (2017). Integrated genomic characterization of oesophageal carcinoma. Nature 541, 169–175. 10.1038/nature20805 28052061 PMC5651175

[B12] Chakrabarti J. Zavros Y. (2020). Generation and use of gastric organoids for the study of *Helicobacter pylori* pathogenesis. Methods Cell. Biol. 159, 23–46. 10.1016/bs.mcb.2020.04.011 32586445

[B13] Cheon D. J. Orsulic S. (2011). Mouse models of cancer. Annu. Rev. Pathol. 6, 95–119. 10.1146/annurev.pathol.3.121806.154244 20936938

[B14] Choi Y. W. Heath E. I. Heitmiller R. Forastiere A. A. Wu T.-T. (2000). Mutations in beta-catenin and APC genes are uncommon in esophageal and esophagogastric junction adenocarcinomas. Mod. Pathol. 13, 1055–1059. 10.1038/modpathol.3880194 11048797

[B15] Co J. Y. Margalef-Català M. Monack D. M. Amieva M. R. (2021). Controlling the polarity of human gastrointestinal organoids to investigate epithelial biology and infectious diseases. Nat. Protoc. 16, 5171–5192. 10.1038/s41596-021-00607-0 34663962 PMC8841224

[B16] Correia A. C. P. Straub D. Read M. Hoefnagel S. J. M. Romero-Pinedo S. Abadía-Molina A. C. (2023). Inhibition of BMP2 and BMP4 represses Barrett's esophagus while enhancing the regeneration of squamous epithelium in preclinical models. Cell. Mol. Gastroenterol. Hepatol. 15, 1199–1217. 10.1016/j.jcmgh.2023.01.003 36706916 PMC10060764

[B17] Costa D. Vene R. Coco S. Longo L. Tosetti F. Scabini S. (2023). SB202190 predicts BRAF-activating mutations in primary colorectal cancer organoids via erk1-2 modulation. Cells 12, 664. 10.3390/cells12040664 36831331 PMC9954675

[B18] Cruz-Acuña R. Kariuki S. W. Sugiura K. Karaiskos S. Plaster E. M. Loebel C. (2023). Engineered hydrogel reveals contribution of matrix mechanics to esophageal adenocarcinoma and identifies matrix-activated therapeutic targets. J. Clin. Investigation 133, e168146. 10.1172/JCI168146 PMC10688988 37788109

[B19] Dekkers J. F. van Vliet E. J. Sachs N. Rosenbluth J. M. Kopper O. Rebel H. G. (2021). Long-term culture, genetic manipulation and xenotransplantation of human normal and breast cancer organoids. Nat. Protoc. 16, 1936–1965. 10.1038/s41596-020-00474-1 33692550 PMC8221035

[B20] Derouet M. F. Allen J. Wilson G. W. Ng C. Radulovich N. Kalimuthu S. (2020). Towards personalized induction therapy for esophageal adenocarcinoma: organoids derived from endoscopic biopsy recapitulate the pre-treatment tumor. Sci. Rep. 10, 14514. 10.1038/s41598-020-71589-4 32884042 PMC7471705

[B21] Dijkstra K. K. Cattaneo C. M. Weeber F. Chalabi M. van de Haar J. Fanchi L. F. (2018) Generation of tumor-reactive T cells by Co-culture of peripheral blood lymphocytes and tumor organoids Cell. 174, 1586–1598.e1512. 10.1016/j.cell.2018.07.009 30100188 PMC6558289

[B22] DiMaio M. A. Kwok S. Montgomery K. D. Lowe A. W. Pai R. K. (2012). Immunohistochemical panel for distinguishing esophageal adenocarcinoma from squamous cell carcinoma: a combination of p63, cytokeratin 5/6, MUC5AC, and anterior gradient homolog 2 allows optimal subtyping. Hum. Pathol. 43, 1799–1807. 10.1016/j.humpath.2012.03.019 22748473 PMC3465493

[B23] Drost J. Karthaus W. R. Gao D. Driehuis E. Sawyers C. L. Chen Y. (2016). Organoid culture systems for prostate epithelial and cancer tissue. Nat. Protoc. 11, 347–358. 10.1038/nprot.2016.006 26797458 PMC4793718

[B24] Drost J. van Jaarsveld R. H. Ponsioen B. Zimberlin C. van Boxtel R. Buijs A. (2015). Sequential cancer mutations in cultured human intestinal stem cells. stem cells Nat. 521, 43–47. 10.1038/nature14415 25924068

[B25] Du L. Wang D. Nagle P. W. Groen A. A. H. Zhang H. Muijs C. T. (2022). Role of mTOR through autophagy in esophageal cancer stemness. Stemness Cancers (Basel) 14, 1806. 10.3390/cancers14071806 35406578 PMC9040713

[B26] Ferlay J. Colombet M. Soerjomataram I. Parkin D. M. Piñeros M. Znaor A. (2021). Cancer statistics for the year 2020: an overview. Int. J. Cancer 149, 778–789. 10.1002/ijc.33588 33818764

[B27] Forsythe S. D. Erali R. A. Sasikumar S. Laney P. Shelkey E. D'Agostino R. Jr. (2021). Organoid platform in preclinical investigation of personalized immunotherapy efficacy in appendiceal cancer: feasibility study. Study Clin. Cancer Res. 27, 5141–5150. 10.1158/1078-0432.Ccr-21-0982 34210684 PMC8720262

[B28] Frankell A. M. Jammula S. Li X. Contino G. Killcoyne S. Abbas S. (2019). The landscape of selection in 551 esophageal adenocarcinomas defines genomic biomarkers for the clinic. Nat. Genet. 51, 506–516. 10.1038/s41588-018-0331-5 30718927 PMC6420087

[B29] Fujii M. Shimokawa M. Date S. Takano A. Matano M. Nanki K. (2016). A colorectal tumor organoid library demonstrates progressive loss of niche factor requirements during tumorigenesis. Stem Cell. 18, 827–838. 10.1016/j.stem.2016.04.003 27212702

[B30] Garcia E. Hayden A. Birts C. Britton E. Cowie A. Pickard K. (2016). Authentication and characterisation of a new oesophageal adenocarcinoma cell line: MFD-1. Sci. Rep. 6, 32417. 10.1038/srep32417 27600491 PMC5013399

[B31] Gotovac J. R. Kader T. Milne J. V. Fujihara K. M. Lara-Gonzalez L. E. Gorringe K. L. (2021). Loss of SMAD4 is sufficient to promote tumorigenesis in a model of dysplastic Barrett's esophagus. Gastroenterology Hepatology 12, 689–713. 10.1016/j.jcmgh.2021.03.008 PMC8267443 33774196

[B32] Hanahan D. (2022). Hallmarks of cancer: new dimensions. Cancer Discov. 12, 31–46. 10.1158/2159-8290.Cd-21-1059 35022204

[B33] Hill D. R. Huang S. Nagy M. S. Yadagiri V. K. Fields C. Mukherjee D. (2017). Bacterial colonization stimulates a complex physiological response in the immature human intestinal epithelium. Elife 6, e29132. 10.7554/eLife.29132 29110754 PMC5711377

[B34] Huang B. Trujillo M. A. Fujikura K. Qiu M. Chen F. Felsenstein M. (2020). Molecular characterization of organoids derived from pancreatic intraductal papillary mucinous neoplasms. J. Pathol. 252, 252–262. 10.1002/path.5515 32696980 PMC8162794

[B35] Huang F. L. Yu S. J. (2018). Esophageal cancer: risk factors, genetic association, and treatment. Asian J. Surg. 41, 210–215. 10.1016/j.asjsur.2016.10.005 27986415

[B36] Hvid-Jensen F. Pedersen L. Drewes A. M. Sørensen H. T. Funch-Jensen P. (2011). Incidence of adenocarcinoma among patients with Barrett's esophagus. N. Engl. J. Med. 365, 1375–1383. 10.1056/NEJMoa1103042 21995385

[B37] Idowu S. Bertrand P. P. Walduck A. K. (2022). Gastric organoids: advancing the study of *H. pylori* pathogenesis and inflammation. Inflamm. Helicobacter 27, e12891. 10.1111/hel.12891 PMC9287064 35384141

[B38] Ilson D. H. van Hillegersberg R. (2018). Management of patients with adenocarcinoma or squamous cancer of the esophagus. Gastroenterology 154, 437–451. 10.1053/j.gastro.2017.09.048 29037469

[B39] Ishizaki T. Uehata M. Tamechika I. Keel J. Nonomura K. Maekawa M. (2000). Pharmacological properties of Y-27632, a specific inhibitor of rho-associated kinases. Mol. Pharmacol. 57, 976–983. 10779382

[B40] Jin R. U. Mills J. C. (2020). Tumor organoids to study gastroesophageal cancer: a primer. a primer J. Mol. Cell. Biol. 12, 593–606. 10.1093/jmcb/mjaa035 32652008 PMC7683018

[B41] Joseph R. R. Yazer E. Hanakawa Y. Stadnyk A. W. (2005). Prostaglandins and activation of AC/cAMP prevents anoikis in IEC-18. Apoptosis 10, 1221–1233. 10.1007/s10495-005-2049-y 16215681

[B42] Jung P. Sato T. Merlos-Suárez A. Barriga F. M. Iglesias M. Rossell D. (2011). Isolation and *in vitro* expansion of human colonic stem cells. stem cells Nat. Med. 17, 1225–1227. 10.1038/nm.2470 21892181

[B43] Kakni P. Jutten B. Teixeira Oliveira Carvalho D. Penders J. Truckenmüller R. Habibovic P. (2023). Hypoxia-tolerant apical-out intestinal organoids to model host-microbiome interactions. J. Tissue Eng. 14, 20417314221149208. 10.1177/20417314221149208 36699634 PMC9869231

[B44] Kamb A. (2005). What's wrong with our cancer models? Nat. Rev. Drug Discov. 4, 161–165. 10.1038/nrd1635 15688078

[B45] Kapoor H. Lohani K. R. Lee T. H. Agrawal D. K. Mittal S. K. (2015). Animal models of Barrett's esophagus and esophageal adenocarcinoma-past, present, and future. Clin. Transl. Sci. 8, 841–847. 10.1111/cts.12304 26211420 PMC4703452

[B46] Karakasheva T. A. Gabre J. T. Sachdeva U. M. Cruz-Acuna R. Lin E. W. DeMarshall M. (2021). Patient-derived organoids as a platform for modeling a patient's response to chemoradiotherapy in esophageal cancer. Sci. Rep. 11, 21304. 10.1038/s41598-021-00706-8 34716381 PMC8556341

[B47] Karakasheva T. A. Kijima T. Shimonosono M. Maekawa H. Sahu V. Gabre J. T. (2020). Generation and characterization of patient-derived head and neck, oral, and esophageal cancer organoids. Cancer Organoids Curr. Protoc. Stem Cell. Biol. 53, e109. 10.1002/cpsc.109 32294323 PMC7350550

[B48] Karthaus W. R. Iaquinta P. J. Drost J. Gracanin A. van Boxtel R. Wongvipat J. (2014) Identification of multipotent luminal progenitor cells in human prostate organoid cultures, Cell 159, 163–175. 10.1016/j.cell.2014.08.017 25201529 PMC4772677

[B49] Kasagi Y. Chandramouleeswaran P. M. Whelan K. A. Tanaka K. Giroux V. Sharma M. (2018). The esophageal organoid system reveals functional interplay between notch and cytokines in reactive Epithelial Changes. Mol. Gastroenterol. Hepatol. 5, 333–352. 10.1016/j.jcmgh.2017.12.013 PMC5852293 29552622

[B50] Kelly R. J. Ajani J. A. Kuzdzal J. Zander T. Van Cutsem E. Piessen G. (2021). Adjuvant nivolumab in resected esophageal or gastroesophageal junction cancer. Cancer N. Engl. J. Med. 384, 1191–1203. 10.1056/NEJMoa2032125 33789008

[B51] Kessler M. Hoffmann K. Brinkmann V. Thieck O. Jackisch S. Toelle B. (2015). The Notch and Wnt pathways regulate stemness and differentiation in human fallopian tube organoids. Nat. Commun. 6, 8989. 10.1038/ncomms9989 26643275 PMC4686873

[B52] Kholosy, W. M. Derieppe M. van den Ham F. Ober K. Su Y. Custers L. (2022). Neuroblastoma and DIPG organoid coculture system for personalized assessment of novel anticancer immunotherapies. J. Personalized Med. 10.3390/jpm11090869 PMC8466534 34575646

[B53] Killcoyne S. Fitzgerald R. C. (2021). Evolution and progression of Barrett's oesophagus to oesophageal cancer. Nat. Rev. Cancer 21, 731–741. 10.1038/s41568-021-00400-x 34545238

[B54] Kinra P. Gahlot G. P. S. Yadav R. Baloda V. Makharia G. K. Gupta S. D. (2018). Histological assessment & use of immunohistochemical markers for detection of dysplasia in Barrett's esophageal mucosa. Pathol. Res. Pract. 214, 993–999. 10.1016/j.prp.2018.05.006 29764708

[B55] Kopper O. de Witte C. J. Lohmussaar K. Valle-Inclan J. E. Hami N. Kester L. (2019). An organoid platform for ovarian cancer captures intra- and interpatient heterogeneity. Nat. Med. 25, 838–849. 10.1038/s41591-019-0422-6 31011202

[B56] Krüger L. Gonzalez L. M. Pridgen T. A. McCall S. J. von Furstenberg R. J. Harnden I. (2017). Ductular and proliferative response of esophageal submucosal glands in a porcine model of esophageal injury and repair. Am. J. Physiol. Gastrointest. Liver Physiol. 313, G180-G191–g191. 10.1152/ajpgi.00036.2017 28572084 PMC5625137

[B57] Kusters J. G. Gerrits M. M. Van Strijp J. A. Vandenbroucke-Grauls C. M. (1997). Coccoid forms of *Helicobacter pylori* are the morphologic manifestation of cell death. Infect. Immun. 65, 3672–3679. 10.1128/iai.65.9.3672-3679.1997 9284136 PMC175523

[B58] Lee N. P. Chan C. M. Tung L. N. Wang H. K. Law S. (2018). Tumor xenograft animal models for esophageal squamous cell carcinoma. J. Biomed. Sci. 25, 66. 10.1186/s12929-018-0468-7 30157855 PMC6116446

[B59] Lee Y. Urbanska A. M. Hayakawa Y. Wang H. Au A. S. Luna A. M. (2017). Gastrin stimulates a cholecystokinin-2-receptor-expressing cardia progenitor cell and promotes progression of Barrett's-like esophagus. Oncotarget 8, 203–214. 10.18632/oncotarget.10667 27448962 PMC5352112

[B60] Li X. Francies H. E. Secrier M. Perner J. Miremadi A. Galeano-Dalmau N. (2018). Organoid cultures recapitulate esophageal adenocarcinoma heterogeneity providing a model for clonality studies and precision therapeutics. Nat. Commun. 9, 2983. 10.1038/s41467-018-05190-9 30061675 PMC6065407

[B61] Liang F. Xu H. Cheng H. Zhao Y. Zhang J. (2023). Patient-derived tumor models: a suitable tool for preclinical studies on esophageal cancer. cancer Cancer Gene Ther. 30, 1443–1455. 10.1038/s41417-023-00652-9 37537209

[B62] Liu D. S. Duong C. P. Phillips W. A. Clemons N. J. (2016). Preclinical models of esophageal adenocarcinoma for drug development. Discov. Med. 22, 371–379. 28147219

[B63] Liu X. Cheng Y. Abraham J. M. Wang Z. Wang Z. Ke X. (2018). Modeling Wnt signaling by CRISPR-Cas9 genome editing recapitulates neoplasia in human Barrett epithelial organoids. Cancer Lett. 436, 109–118. 10.1016/j.canlet.2018.08.017 30144514 PMC6152930

[B64] Maenhoudt N. Defraye C. Boretto M. Jan Z. Heremans R. Boeckx B. (2020). Developing organoids from ovarian cancer as experimental and preclinical models. Stem Cell. Rep. 14, 717–729. 10.1016/j.stemcr.2020.03.004 PMC7160357 32243841

[B65] Mahmoudian R. A. Farshchian M. Abbaszadegan M. R. (2021). Genetically engineered mouse models of esophageal cancer. Exp. Cell. Res. 406, 112757. 10.1016/j.yexcr.2021.112757 34331909

[B66] Maimets M. Rocchi C. Bron R. Pringle S. Kuipers J. Ben N. G. (2016). Long-term In Vitro expansion of salivary gland stem cells driven by Wnt signals. Stem Cell. Rep. 6, 150–162. 10.1016/j.stemcr.2015.11.009 PMC4720006 26724906

[B67] Matano M. Date S. Shimokawa M. Takano A. Fujii M. Ohta Y. (2015). Modeling colorectal cancer using CRISPR-Cas9-mediated engineering of human intestinal organoids. Nat. Med. 21, 256–262. 10.1038/nm.3802 25706875

[B68] Matson V. Chervin C. S. Gajewski T. F. (2021). Cancer and the Microbiome: influence of the commensal microbiota on cancer, immune responses, and immunotherapy. Immunother. Gastroenterol. 160, 600–613. 10.1053/j.gastro.2020.11.041 PMC8409239 33253684

[B69] Mazzucchelli S. Piccotti F. Allevi R. Truffi M. Sorrentino L. Russo L. (2019). Establishment and morphological characterization of patient-derived organoids from breast cancer. Breast Cancer Biol. Proced. Online 21, 12. 10.1186/s12575-019-0099-8 31223292 PMC6570967

[B70] Mishra L. Shetty K. Tang Y. Stuart A. Byers S. W. (2005). The role of TGF-beta and Wnt signaling in gastrointestinal stem cells and cancer. cancer Oncogene 24, 5775–5789. 10.1038/sj.onc.1208924 16123810

[B71] Moss S. F. (2017). The clinical evidence linking *Helicobacter pylori* to gastric cancer. Gastroenterology Hepatology 3, 183–191. 10.1016/j.jcmgh.2016.12.001 PMC5331857 28275685

[B72] Naruse M. Masui R. Ochiai M. Maru Y. Hippo Y. Imai T. (2020). An organoid-based carcinogenesis model induced by *in vitro* chemical treatment. Carcinogenesis 41, 1444–1453. 10.1093/carcin/bgaa011 32047892

[B73] Neal J. T. Li X. Zhu J. Giangarra V. Grzeskowiak C. L. Ju J. (2018) Organoid modeling of the tumor immune microenvironment Cell, 175, 1972–1988. 10.1016/j.cell.2018.11.021 30550791 PMC6656687

[B74] Newell F. Patel K. Gartside M. Krause L. Brosda S. Aoude L. G. (2019). Complex structural rearrangements are present in high-grade dysplastic Barrett's oesophagus samples. BMC Med. Genomics 12, 31. 10.1186/s12920-019-0476-9 30717762 PMC6360790

[B75] Njei B. McCarty T. R. Birk J. W. (2016). Trends in esophageal cancer survival in United States adults from 1973 to 2009: a SEER database analysis. J. Gastroenterology Hepatology 31, 1141–1146. 10.1111/jgh.13289 PMC4885788 26749521

[B76] Nowicki-Osuch K. Zhuang L. Jammula S. Bleaney C. W. Mahbubani K. T. Devonshire G. (2021) Molecular phenotyping reveals the identity of Barrett's esophagus and its malignant transition Science, 373, 760–767. 10.1126/science.abd1449 34385390

[B77] Obermannová R. Alsina M. Cervantes A. Leong T. Lordick F. Nilsson M. (2022). Oesophageal cancer: ESMO Clinical Practice Guideline for diagnosis, treatment and follow-up. Ann. Oncol. 33, 992–1004. 10.1016/j.annonc.2022.07.003 35914638

[B78] Osaki L. H. Gama P. (2013). MAPKs and signal transduction in the control of gastrointestinal epithelial cell proliferation and differentiation. Int. J. Mol. Sci. 14, 10143–10161. 10.3390/ijms140510143 23670595 PMC3676833

[B79] Polyzos S. A. Zeglinas C. Artemaki F. Doulberis M. Kazakos E. Katsinelos P. (2018). *Helicobacter pylori* infection and esophageal adenocarcinoma: a review and a personal view. Ann. Gastroenterol. 31, 8–13. 10.20524/aog.2017.0213 29333062 PMC5759616

[B80] Porter R. J. Murray G. I. McLean M. H. (2020). Current concepts in tumour-derived organoids. Br. J. Cancer 123, 1209–1218. 10.1038/s41416-020-0993-5 32728094 PMC7555542

[B81] Puschhof J. Pleguezuelos-Manzano C. Martinez-Silgado A. Akkerman N. Saftien A. Boot C. (2021). Intestinal organoid cocultures with microbes. Nat. Protoc. 16, 4633–4649. 10.1038/s41596-021-00589-z 34381208

[B82] Quante M. Bhagat G. Abrams J. A. Marache F. Good P. Lee M. D. (2012). Bile acid and inflammation activate gastric cardia stem cells in a mouse model of Barrett-like metaplasia. Cancer Cell. 21, 36–51. 10.1016/j.ccr.2011.12.004 22264787 PMC3266546

[B83] Que J. Garman K. S. Souza R. F. Spechler S. J. (2019) Pathogenesis and cells of origin of Barrett's esophagus Gastroenterology 157, 349–364. 10.1053/j.gastro.2019.03.072 31082367 PMC6650338

[B84] Sachs N. de Ligt J. Kopper O. Gogola E. Bounova G. Weeber F. (2018). A living biobank of breast cancer organoids captures disease heterogeneity. Cell. 172, 373–386. 10.1016/j.cell.2017.11.010 29224780

[B85] Sato T. Stange D. E. Ferrante M. Vries R. G. Van Es J. H. Van den Brink S. (2011). Long-term expansion of epithelial organoids from human colon, adenoma, adenocarcinoma, and Barrett's epithelium. s epithelium Gastroenterol. 141, 1762–1772. 10.1053/j.gastro.2011.07.050 21889923

[B86] Sato T. Vries R. G. Snippert H. J. van de Wetering M. Barker N. Stange D. E. (2009a). Single Lgr5 stem cells build crypt-villus structures *in vitro* without a mesenchymal niche. Nature 459, 262–265. 10.1038/nature07935 19329995

[B87] Schepers A. Clevers H. (2012). Wnt signaling, stem cells, and cancer of the gastrointestinal tract. Cold Spring Harb. Perspect. Biol. 4, a007989. 10.1101/cshperspect.a007989 22474007 PMC3312683

[B88] Secrier M. Li X. de Silva N. Eldridge M. D. Contino G. Bornschein J. (2016a). Mutational signatures in esophageal adenocarcinoma define etiologically distinct subgroups with therapeutic relevance. Nat. Genet. 48, 1131–1141. 10.1038/ng.3659 27595477 PMC5957269

[B89] Segal E. D. Falkow S. Tompkins L. S. (1996). *Helicobacter pylori* attachment to gastric cells induces cytoskeletal rearrangements and tyrosine phosphorylation of host cell proteins. Proc. Natl. Acad. Sci. U. S. A. 93, 1259–1264. 10.1073/pnas.93.3.1259 8577751 PMC40067

[B90] Sepich-Poore G. D. Zitvogel L. Straussman R. Hasty J. Wargo J. A. Knight R. (2021). The microbiome and human cancer. cancer Sci. 371, eabc4552. 10.1126/science.abc4552 PMC8767999 33766858

[B91] Smit J. K. Faber H. Niemantsverdriet M. Baanstra M. Bussink J. Hollema H. (2013). Prediction of response to radiotherapy in the treatment of esophageal cancer using stem cell markers. Radiotherapy Oncol. 107, 434–441. 10.1016/j.radonc.2013.03.027 23684587

[B92] Song S. Ajani J. A. Honjo S. Maru D. M. Chen Q. Scott A. W. (2014). Hippo coactivator YAP1 upregulates SOX9 and endows esophageal cancer cells with stem-like properties. Cancer Res. 74, 4170–4182. 10.1158/0008-5472.Can-13-3569 24906622 PMC4136429

[B93] Stanforth K. Chater P. Brownlee I. Wilcox M. Ward C. Pearson J. (2021). *In vitro* modelling of the mucosa of the oesophagus and upper digestive tract: narrative review Annals of Esophagus. Available at: https://aoe.amegroups.org/article/view/5958 .

[B94] Tao Y. Kang B. Petkovich D. A. Bhandari Y. R. In J. Stein-O'Brien G. (2019) Aging-like spontaneous epigenetic silencing facilitates Wnt activation, Stemness, Braf(V600E)-Induced Tumorigenesis Cancer Cell. 35, 315–328.e31. 10.1016/j.ccell.2019.01.005 30753828 PMC6636642

[B95] Theisen J. Stein H. J. Dittler H. J. Feith M. Moebius C. Kauer W. K. (2002). Preoperative chemotherapy unmasks underlying Barrett's mucosa in patients with adenocarcinoma of the distal esophagus. Surg. Endosc. 16, 671–673. 10.1007/s00464-001-8307-3 11972212

[B96] Turco M. Y. Gardner L. Hughes J. Cindrova-Davies T. Gomez M. J. Farrell L. (2017). Long-term, hormone-responsive organoid cultures of human endometrium in a chemically defined medium. Nat. Cell. Biol. 19, 568–577. 10.1038/ncb3516 28394884 PMC5410172

[B97] Uhlenhopp D. J. Then E. O. Sunkara T. Gaduputi V. (2020). Epidemiology of esophageal cancer: update in global trends, etiology and risk factors. J. Gastroenterology 13, 1010–1021. 10.1007/s12328-020-01237-x 32965635

[B98] Underwood T. J. Derouet M. F. White M. J. Noble F. Moutasim K. A. Smith E. (2010). A comparison of primary oesophageal squamous epithelial cells with HET-1A in organotypic culture. Biol. Cell. 102, 635–644. 10.1042/bc20100071 20843300

[B99] van de Wetering M. Francies H. E. Francis J. M. Bounova G. Iorio F. Pronk A. (2015). Prospective derivation of a living organoid biobank of colorectal cancer patients. Cell. 161, 933–945. 10.1016/j.cell.2015.03.053 25957691 PMC6428276

[B100] VanDussen K. L. Marinshaw J. M. Shaikh N. Miyoshi H. Moon C. Tarr P. I. (2015). Development of an enhanced human gastrointestinal epithelial culture system to facilitate patient-based assays. Gut 64, 911–920. 10.1136/gutjnl-2013-306651 25007816 PMC4305344

[B101] Van Nieuwenhove Y. Willems G. (1998). Gastroesophageal reflux triggers proliferative activity of the submucosal glands in the canine esophagus. Dis. Esophagus 11, 89–93. 10.1093/dote/11.2.89 9779363

[B102] Vlachogiannis G. Hedayat S. Vatsiou A. Jamin Y. Fernández-Mateos J. Khan K. (2018) Patient-derived organoids model treatment response of metastatic gastrointestinal cancers Science, 359, 920–926. 10.1126/science.aao2774 29472484 PMC6112415

[B103] Vogelstein B. Fearon E. R. Hamilton S. R. Kern S. E. Preisinger A. C. Leppert M. (1988). Genetic alterations during colorectal-tumor development. Dev. N. Engl. J. Med. 319, 525–532. 10.1056/NEJM198809013190901 2841597

[B104] Wang D. H. Clemons N. J. Miyashita T. Dupuy A. J. Zhang W. Szczepny A. (2010). Aberrant epithelial-mesenchymal Hedgehog signaling characterizes Barrett's metaplasia. Gastroenterology 138, 1810–1822. 10.1053/j.gastro.2010.01.048 20138038 PMC3422577

[B105] Wang J. L. Yu J. P. Sun Z. Q. Sun S. P. (2014). Radiobiological characteristics of cancer stem cells from esophageal cancer cell lines. World J. Gastroenterol. 20, 18296–18305. 10.3748/wjg.v20.i48.18296 25561796 PMC4277966

[B106] Westra W. M. Straub D. Milano F. Buttar N. S. Wang K. K. Krishnadath K. K. (2022). Inhibition of the BMP pathway prevents development of Barrett's-associated adenocarcinoma in a surgical rat model. Dis. Esophagus 35, doab072. 10.1093/dote/doab072 34718471 PMC9113020

[B107] Yan H. H. N. Siu H. C. Law S. Ho S. L. Yue S. S. K. Tsui W. Y. (2018). A comprehensive human gastric cancer organoid biobank captures tumor subtype heterogeneity and enables therapeutic screening. Cell. Stem Cell. 23, 882–897. 10.1016/j.stem.2018.09.016 30344100

[B108] Yu L. Li Z. Mei H. Li W. Chen D. Liu L. (2021). Patient-derived organoids of bladder cancer recapitulate antigen expression profiles and serve as a personal evaluation model for CAR-T cells *in vitro* . Immunology 10, e1248. 10.1002/cti2.1248 PMC7847802 33552510

[B109] Zanconato F. Cordenonsi M. Piccolo S. (2016). YAP/TAZ at the roots of cancer. Cancer Cancer Cell. 29, 783–803. 10.1016/j.ccell.2016.05.005 27300434 PMC6186419

[B110] Zarubin T. Han J. (2005). Activation and signaling of the p38 MAP kinase pathway. Cell. Res. 15, 11–18. 10.1038/sj.cr.7290257 15686620

[B111] Zhang Q. Bansal A. Dunbar K. B. Chang Y. Zhang J. Balaji U. (2022). A human Barrett's esophagus organoid system reveals epithelial-mesenchymal plasticity induced by acid and bile salts. Am. J. Physiol. Gastrointest. Liver Physiol. 322, G598–g614. 10.1152/ajpgi.00017.2022 35380457 PMC9109796

[B112] Zhang Y. Yang Y. Jiang M. Huang S. X. Zhang W. Al Alam D. (2018). 3D modeling of esophageal development using human PSC-derived basal progenitors reveals a critical role for notch signaling cell. Stem Cell. 23, 516–529.e515. 10.1016/j.stem.2018.08.009 PMC6282026 30244870

[B113] Zhao H. Cheng Y. Kalra A. Ma K. Zheng Y. Ziman B. (2022). Generation and multiomic profiling of a TP53/CDKN2A double-knockout gastroesophageal junction organoid model. Sci. Transl. Med. 14, eabq6146. 10.1126/scitranslmed.abq6146 36449602 PMC10026384

